# Effects of adaptive left bundle branch–optimized cardiac resynchronization therapy: a single centre experience

**DOI:** 10.1186/s12872-022-02742-2

**Published:** 2022-08-06

**Authors:** Xiang-Fei Feng, Ling-Chao Yang, Yan Zhao, Yi-Chi Yu, Bo Liu, Yi-Gang Li

**Affiliations:** Department of Cardiology, School of Medicine, Xinhua Hospital, Shanghai Jiao Tong University, #1665, KongJiang Road, Shanghai, 200092 China

**Keywords:** Cardiac resynchronization therapy, Left bundle branch block, Left bundle branch area pacing, Heart failure, Ischaemic cardiomyopathy

## Abstract

**Background:**

Adaptive cardiac resynchronization therapy (aCRT) is associated with improved clinical outcomes. Left bundle branch area pacing (LBBAP) has shown encouraging results as an alternative option for aCRT. A technique that can be accomplished effectively using LBBAP combined with coronary venous pacing (LOT-aCRT). We aimed to assess the feasibility and outcomes of LOT-aCRT.

**Methods:**

LOT-aCRT, capable of providing two pacing modes, LBBAP alone or LBBAP combined with LV pacing, was attempted in patients with CRT indications. Patients were divided into two groups: those with LBBAP and LV pacing (LOT-aCRT) and those with conventional biventricular pacing (BVP-aCRT).

**Results:**

A total of 21 patients were enrolled in the study (10 in the LOT-aCRT group, 11 in the BVP-aCRT group). In the LOT-aCRT group, the QRS duration (QRSd) via BVP was narrowed from 158.0 ± 13.0 ms at baseline to 132.0 ± 4.5 ms (*P* = 0.019) during the procedure, and further narrowed to 123.0 ± 5.7 ms (*P* < 0.01) via LBBAP. After the procedure, when LOT-aCRT implanted and worked, QRSd was further changed to 121.0 ± 3.8 ms, but the change was not significant (*P* > 0.05). In the BVP-aCRT group, BVP resulted in a significant reduction in the QRSd from 176.7 ± 19.7 ms at baseline to 133.3 ± 8.2 ms (*P* = 0.011). However, compared with LOT-aCRT, BVP has no advantage in reducing QRSd and the difference was statistically significant (*P* < 0.01). During 9 months of follow-up, patients in both groups showed improvements in the LVEF and NT-proBNP levels (all *P* < 0.01). However, compared with BVP-aCRT, LOT-aCRT showed more significant changes in these parameters (*P* < 0.01).

**Conclusions:**

The study demonstrates that LOT-aCRT is clinically feasible in patients with systolic heart failure and LBBB. LOT-aCRT was associated with significant narrowing of the QRSd and improvement in LV function.

## Introduction

Cardiac resynchronization therapy (CRT) with biventricular pacing (BVP) is an established therapy for symptomatic heart failure (HF) patients with left ventricular systolic dysfunction and a wide QRS complex, particularly left bundle branch block (LBBB) [1, 2]. However, up to one-third of patients treated with BVP-CRT are nonresponders [3]. There are multiple reasons for this nonresponse, including left ventricular (LV) scar burden and distribution, suboptimal LV stimulation site, sex, and limited electrical or mechanical dyssynchrony [4]. There is evidence that CRT is not salutary in patients with posterolateral scarring [5].

Recently, left bundle branch area pacing (LBBAP) has shown potential in restoring physiological activation by engaging the intrinsic His-Purkinje system [6, 7]. Initial investigations have shown that this technique can provide a relatively narrow QRS duration (QRSd), LBBB correction, and a low and stable pacing output [4, 8]. Some studies have further demonstrated that this technique is clinically feasible in patients with systolic HF and LBBB and is currently considered a viable alternative to BVP-CRT in patients requiring CRT [7, 9].

However, in patients with distal conduction delay in the distal LBB, LV Purkinje network, or myocardium, left bundle branch pacing is inherently limited in its ability to restore physiological activation [10]. Moreover, in patients with an atypical LBBB morphology, LBBAP could only achieve partial reduction of the QRSd [11]. Combining LBBAP and BVP pacing might address some of the above mentioned limitations of both techniques, especially in patients with more advanced heart failure [10].

**Fig. 1:**
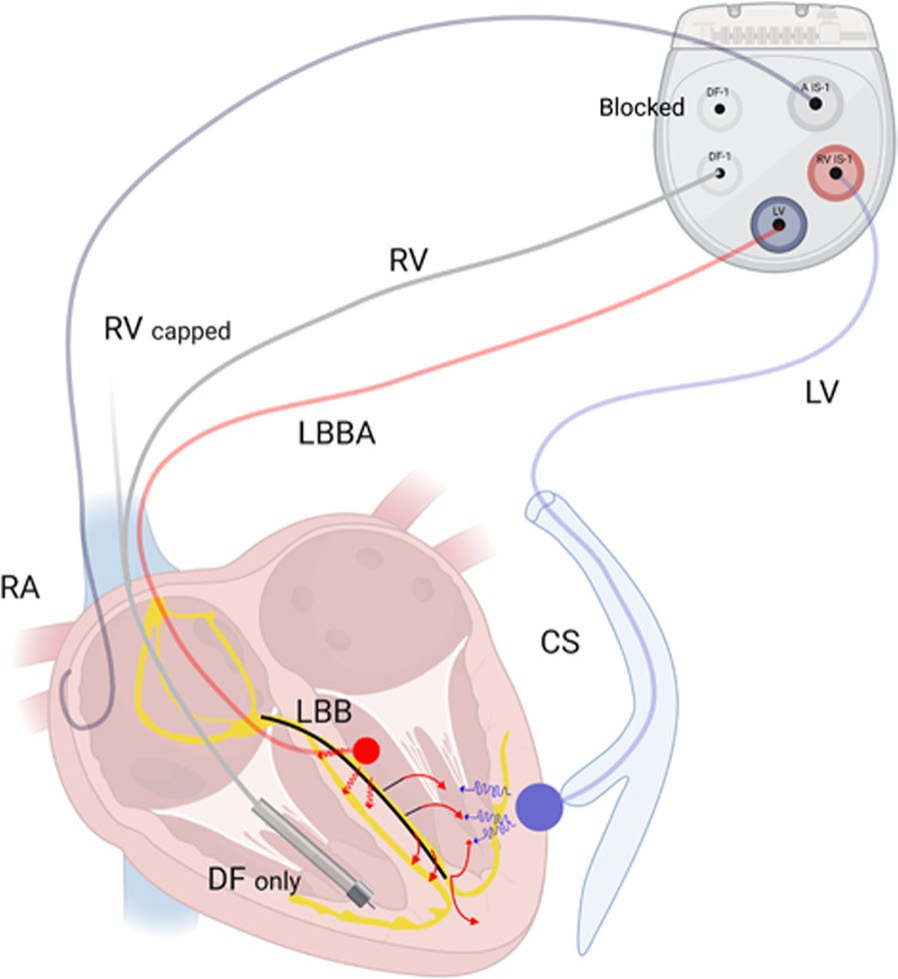
Schematic of device connections in the LOT-aCRTD procedure. *LV* coronary venous lead, *DF* defibrillation, *RV* right ventricle lead, LBBA, left bundle branch area lead, *CS* coronary venous, *RA* right atrial lead

We developed a technique that can be accomplished effectively using LBBAP followed by sequential coronary venous (CS) pacing [9] (Fig. 1). A novel adaptive CRT (aCRT) algorithm that provides LV pacing alone when AV conduction is normal and BV pacing when AV conduction is prolonged, demonstrated the noninferiority of the aCRT algorithm compared to echo-guided BVP [12]. The combination of adaptive CRT and LBBAP (LOT-aCRT) is a truly innovative method in this paper, capable of providing two pacing modes, LBBAP alone or LBBAP combined with LV pacing. It should theoretically achieve optimal CRT effects in nonresponders.

Patients with atypical LBBB and a higher overall scar burden, especially in ischaemic cardiomyopathy (ICM), might be the desired candidates for this procedure. However the outcome and the effects of LOT-aCRT are still unknown. The objective of this study was to assess the clinical feasibility, safety, and efficacy of LOT-aCRT in patients with HF and LBBB.

## Methods

This prospective observational single centre study enrolled all patients with CRT indications between February 1, 2019 and June 30, 2021. The choice of LBBAP was based on the patient’s discretion. Patients were divided into two groups: those with LBBAP and LV pacing (LOT-aCRT, group 1) and those with conventional biventricular pacing (BVP-aCRT, group 2). To reduce selection bias, we only selected currently available models (DTBA2D1, DTBA2D4, and C5TR01) for aCRT from Medtronic Inc. (Minneapolis, USA).

This study was approved by the Ethics Committee of Xinhua Hospital Affiliated with Shanghai Jiao Tong University, School of Medicine (approval number: XHEC-D-2020-148) and performed in accordance with the Declaration of Helsinki.

### Study subjects

Patients with drug-refractory New York Heart Association classes II to IV HF symptoms, left ventricular ejection fraction (LVEF) ≤ 35%, LBBB (QRS > 130 ms) or QRS > 150 ms (ms) without LBBB were eligible for CRT [13]. According to the Strauss criteria [14], patients with preserved AV conduction and LBBB morphology were selected first for adaptive CRT. Intrinsic preserved AV conduction was defined as a PR interval ≤ 200 ms as documented on an at-rest 12-lead ECG [15].

Patients were excluded if they had right bundle branch block (RBBB), chronic atrial fibrillation, use of a left ventricular assist device, metastatic cancer, or a life expectancy less than 1 year. Written informed consent was obtained from each patient.

### Procedural details

The right ventricular defibrillator lead was first implanted in the right ventricle (RV) to provide backup ventricular pacing if the patient developed transient complete atrioventricular block during LBBAP lead placement. Subsequently, the coronary sinus (CS) lead was implanted using routine implantation techniques, and targeting sites were determined according to the maximal LV delay [16, 17]. Then, LBBAP was performed using the Select Secure pacing lead. All defibrillator electrodes were implanted in the RV apical position. The atrial lead was placed at the right atrial appendage (Fig. 1). The fluoroscopy durations for the entire procedure, LBBAP lead implantation and LV lead implantation were separately recorded.

### LBBAP lead implantation technique

As previously described [8, 18–20], a Select Site C315 His sheath and a Select Secure 3830 pacing lead (Medtronic Inc., USA) were advanced to the implantation site. The right ventricular septal location for LBBAP was identified using the anatomical location and pacing localization of the nine-grid system [21]. Once the implantation site was identified, the pacing lead was advanced deep into the septum while the unipolar pacing impedance, electrogram characteristics and paced QRS morphology were monitored.

Additionally, the lead orientation was displayed in various projections. Generally, the sheath and the lead were oriented gently and the lead should point to the 12- to 1-o’clock direction from a right anterior oblique viewing angle of 30° and the 2- to 3-o’clock direction from a left anterior oblique viewing angle of 30° [22].

Successful LBBAP was characterized as capturing the LBB with or without myocardial capture, with a narrow RBBB morphology [19, 21]. If an acceptable LBB capture could not be achieved after 5 attempts of lead positioning, it was considered procedure failure [23].

### Optimal CS location

The details of the device and procedure have been described elsewhere [16, 17]. Optimal vein selection and lead implantation were greatly facilitated by high-quality occlusive venography. Traditionally, CS intubation was performed by advancing a 0.035-inch hydrophilic wire to the region of the CS ostium via a preformed guide catheter and probing to locate the CS ostium. Venograms were typically performed in the anteroposterior and left anterior oblique projections. The optimal CS location was limited to the distribution of the coronary veins [16, 17].

### Intraoperative measurements

Intraoperative lead testing included R waves, impedance, and the pace threshold at 0.4 ms. For both group 1 and group 2, the morphology and duration of the QRS waves at baseline and during LBBAP, CS pacing, and BVP (RV defibrillator lead and CS lead) were measured on the EP recording system at 100 mm/s. Left ventricular activation time (LVAT) during LBBAP was documented, which was defined as the time from the stimulus onset to the peak of the R wave [24].

### Device Connection

All RV defibrillator leads were single coil lead and had 2 connector pins, one for pace/sense (IS-1), and the other for high voltage defibrillation (DF-1). The pace/sense connector pin was capped (Fig. 1). All atrial leads were implanted and connected to the A port.

In group 1, the patients underwent CRT-defibrillator (CRTD) treatment (model DTBA2D1), the CS lead was connected to the pace-sensing portion of the RV port, and the LBBAP lead was connected to the LV port. The SVC port (DF-1) was blocked. In patients undergoing CRT-pacemaker (CRTP) treatment (model C5TR01), the LBBAP lead was connected to the LV port. The CS lead was still connected to the RV port.

In group 2, the patients underwent CRTD treatment (model DTBA2D4), and the CS lead was connected to the LV port. Then the RV defibrillator lead was connected to the RV port.

### Programming and follow up

Before hospital discharge, separate “zones” could be programmed for detection of ventricular fibrillation (VF) and ventricular tachycardia (VT). All patients were seen for routine clinical follow-up at standard time intervals (every 3 months). Functional status was assessed by the NYHA classification system. Device thresholds were checked and adjusted as needed to maximize battery longevity. The pacing threshold, impedance and R wave amplitude were measured. All device-detected and treated VT/VF episodes were reviewed and adjudicated by an independent episode reviewer.

LBBAP was set to bipolar pacing with a 0.4 ms pulse width in all patients. According to previous literature [25], a high pacing threshold was defined as a pacing threshold over 2.5 V/0.4 ms or an increase of more than 1.0 V compared with the baseline after the procedure and at follow-up. Echocardiographic indices, including LVEF, LV end-diastolic dimension (LVEDD), and pulmonary artery systolic pressure, were recorded before implantation and at follow-up.

### The aCRT algorithm

The details of the aCRT algorithm were published previously [12]. If the conduction interval from the right atrium to the right ventricle is normal (intrinsic AV ≤ 200 ms if in sinus rhythm, or AV ≤ 250 ms, if receiving atrial pacing) and the heart rate does not exceed 100 beats/min, the algorithm provides synchronized LV pacing [15]. Conversely, if the intrinsic AV conduction interval is prolonged, the algorithm provides BV pacing. The QRSd values via LBB-optimized LV pacing were measured.

In group 1, where the CS lead is connected to the RV channel and LABBAP is connected to the LV channel, “LV only” is suggested by the aCRT algorithm, which means LBBAP only.

### Statistical analysis

The data were entered into a database formulated within the SPSS data management system (SPSS 18.0). Continuous variables were presented as the mean ± SD, and compared using a Student’s t test. Categorical variables are expressed as proportions, and differences between the groups were assessed using the *χ*^2^ test. Differences in subgroups were assessed using ANOVA. A two-sided *P* value of < 0.05 was considered statistically significant. When writing the report, the STROBE checklist was used [26].

## Results

During the study period, 26 patients underwent CRT procedures. Four of them were excluded according to the exclusion criteria. During follow-up, one patient in group 1 was lost to follow-up. Therefore a total of 21 patients were enrolled in the study (10 in group 1, 11 in group 2). All patients had preserved AV conduction and had at least 1 HF hospitalization within 3 months before CRT/D implantation. Entresto (sacubitril/valsartan), β-blockers, and loop diuretics were prescribed to all patients.

### Baseline characteristics

Among the 21 patients, nine (42.9%) were male. All patients had cardiomyopathy (10 nonischaemic and 11 ischaemic), and 7 patients had paroxysmal atrial fibrillation. Hypertension was present in 10 patients. Frequent ventricular premature contraction (VPC) (> 1000 per 24 h [27]) was found in 6 patients. The mean age was 69.1 ± 6.4 years, and the baseline characteristics of the patients are provided in Table 1. At baseline, the two groups were matched for age, sex, hypertension, diabetes mellitus, ICM, and paroxysmal atrial fibrillation as illustrated in Table 1 (all *P* > 0.05).

**Table 1:** Baseline characteristics of the 21 patients with a CRT-D/P (n = 21)

	Total (n = 21)	Group 1 (n = 10)	Group 2 (n = 11)	*P* value
Age (years)	69.1 ± 6.4	71.8 ± 5.1	66.8 ± 6.9	0.217
Sex, Male, n (%)	9 (42.9%)	4 (40.0%)	5 (45.5%)	1.000
Diabetes mellitus, n (%)	4 (19.0%)	2 (20.0%)	2 (18.2%)	1.000
Hypertension, n (%)	10 (47.6%)	5 (50.0%)	5 (45.5%)	1.000
Frequent VPC, n (%)	6 (28.6%)	2 (20.0%)	4 (36.4%)	0.730
ICM, n (%)	11 (52.4%)	6 (60.0%)	5 (45.5%)	0.819
PCI, n (%)	11 (52.4%)	6 (60.0%)	5 (45.5%)	0.819
NT-proBNP (pg/ml)	2937 ± 1646	3240 ± 2258	2684 ± 1083	0.634
LVEF (%)	33.1 ± 3.0	32.0 ± 4.2	34.0 ± 1.3	0.302
PAF, n (%)	7 (33.3%)	4 (40.0%)	3 (27.3%)	0.877

*NT-proBNP* N-terminal pro B type brain natriuretic peptide, *LVEF* left ventricular ejection fraction, *PCI* percutaneous transluminal coronary intervention, *VPC* ventricular premature contraction, *PAF* paroxysmal atrial fibrillation, *ICM* ischaemic cardiomyopathy

**Fig. 2:**
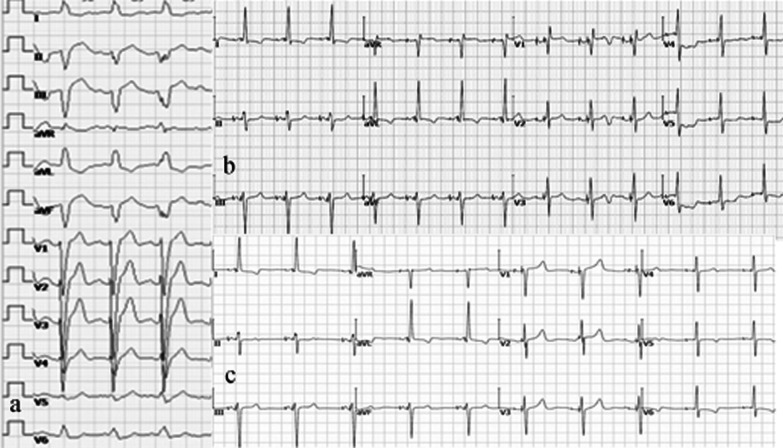
ECG following LOT-aCRTD in a patient with ischaemic cardiomyopathy and normal PR interval. **a** Baseline ECG shows LBBB with a QRS duration of 160 ms. **b** During unipolar LBBAP pacing, a right bundle branch block pattern with a QRS duration of 122 ms is visible. **c** During pacing with LOT-aCRTD, a left bundle branch block correction pattern with a QRS duration of 120 ms is visible

The echocardiographic indices, including LVEF, LVEDD, NYHA classification, and NT-proBNP are shown in Table 3. Baseline parameters were similar between the two groups (all *P* > 0.05). The baseline LVEF and the baseline QRSd (Fig. 2a) were 33.9 ± 3.9% and 168.2 ± 18.9 ms, respectively. At baseline, the QRSd of the two groups were matched (158.0 ± 13.0, vs. 176.7 ± 19.7, *P* > 0.05).

### Procedural Outcomes

**Fig. 3:**
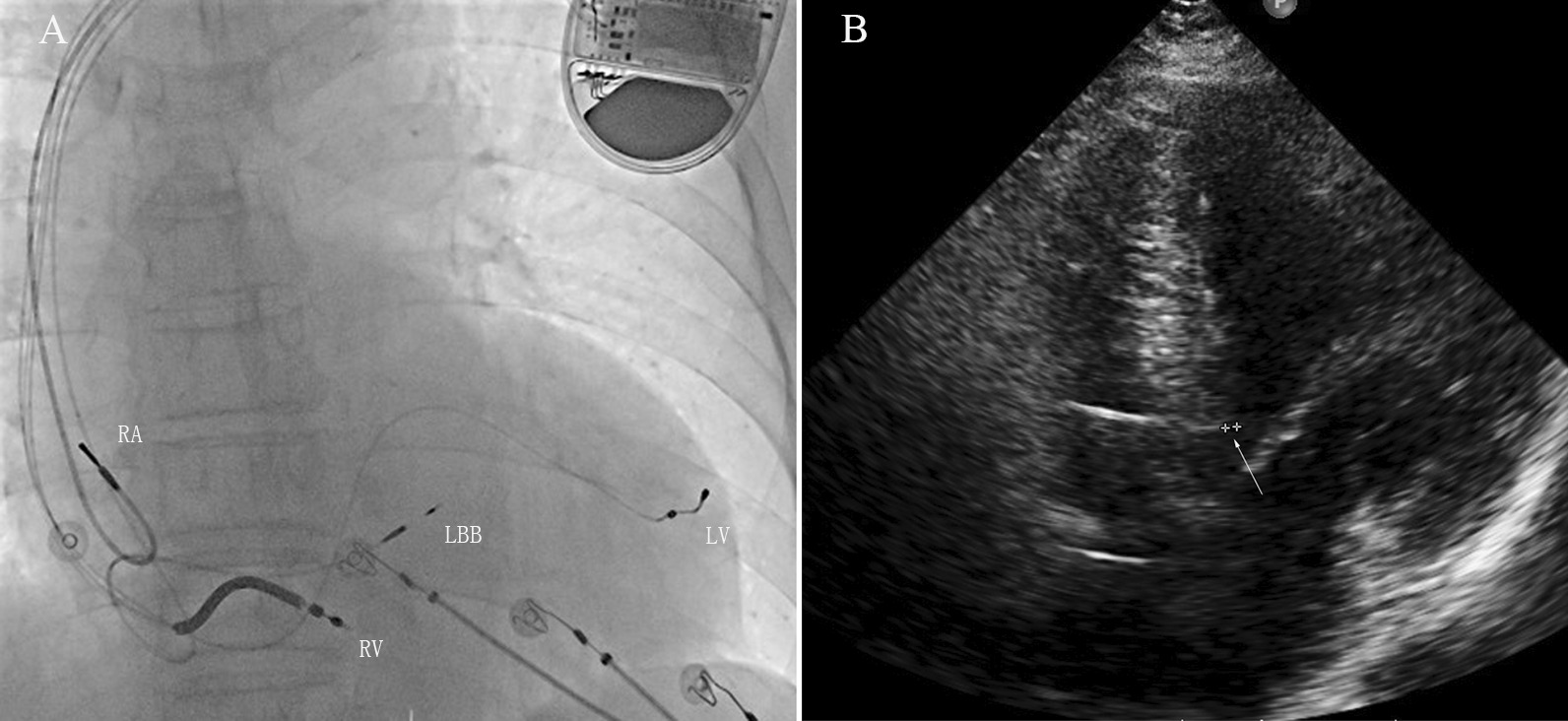
Fluoroscopic image and echo image of an LOT-aCRTD in a patient with ischaemic cardiomyopathy and a normal PR interval. **a** Fluoroscopic image in the RAO 30° projections. This image shows the final LBB lead position in the interventricular septum. *RA* right atrial lead, *LV* coronary sinus lead, *LBB* left bundle branch lead, *RV* right ventricular defibrillator lead. **b** Transthoracic echocardiogram image. The apical four-chamber view demonstrates the depth of the LBB lead in the interventricular septum (arrow)

CRTDs were implanted in 19 patients (Fig. 3a), and CRTPs were implanted in the remaining 2 patients, one in each group. The operation duration was 135 ± 26 min. The duration of X-ray fluoroscopy was 25.2 ± 7.1 min.

In group 1, the LBBA lead was successfully implanted in 9 patients, and the acute success rate was 90.0%. Another patient received LV septum pacing with a paced QRSd > 130 ms after LBBAP failed 5 times. In group 2, the CS lead and RV defibrillation lead were successfully implanted in all 11 patients. Compared with those of group 2, the operation duration was significantly prolonged and the duration of X-ray fluoroscopy tended to be longer in group 1 (Table 2).

**Table 2:** Procedural characteristics in patients with a CRT-D/P (mean ± SD) (n = 21)

	Total (n = 21)	Group 1 (n = 10)	Group 2 (n = 11)	*P* value
*LBBA lead*				
R-wave amplitude	–	9.9 ± 7.2	–	–
Threshold (unipolar) (V/0.4 ms)	–	0.84 ± 0.17	–	–
Impedance (unipolar) (Ω)	–	678 ± 102	–	–
LVAT (ms)	–	75.2 ± 9.4	–	–
*RV* *DF lead*				
R-wave amplitude	23.5 ± 8.4	24.3 ± 11.8	23.0 ± 6.5	0.825
Threshold (unipolar) (V/0.4 ms)	0.82 ± 0.20	0.93 ± 0.10	0.75 ± 0.23	0.187
Impedance (unipolar) (Ω)	578 ± 147	626 ± 77	546 ± 180	0.434
*CS lead*				
R-wave amplitude	18.3 ± 9.4	13.8 ± 2.6	22.1 ± 11.6	0.145
Threshold (unipolar) (V/0.4 ms)	1.0 ± 0.24	0.96 ± 0.27	1.12 ± 0.20	0.301
Impedance (unipolar) (Ω)	708 ± 134	745 ± 97	678 ± 160	0.434
CRTD (%)	19(90.5%)	9(90.0%)	10(90.9%)	1.000
Fluoroscopy Time (min)	25.2 ± 7.1	29.2 ± 8.8	21.8 ± 3.1	0.086
Procedure time (min)	135 ± 26	152 ± 31	122 ± 10	0.04

*LBBA* left bundle branch area, *LV* left ventricle, *RV* right ventricle, *DF* defibrillation, *CS* coronary venous, *LVAT* left ventricular activation time

Neither group showed differences in CS pacing lead, RV defibrillator lead parameters, such as R-wave amplitude, threshold, and impedance (Table 2). Both the LBBAP and CS capture thresholds remained stable during the procedure (1.3 ± 0.6 V at 0.4 ms vs. 1.6 ± 0.7 V at 0.4 ms).

During the procedure, temporary RBBB and acute perforation of the ventricular septum were documented in 1 patient each in group 1. The lead was successfully repositioned and no pericardial effusion or cerebral ischaemia was observed. In group 2, no complications were documented.

### ECG characteristics and pacing parameters

Individual electrocardiographic responses to RV, CS, and LBBAP at the time of implantation are shown in Table 2. Among the 21 patients, the baseline QRSd was 168.1 ± 18.9 ms (Fig. 2a).

In group 1, after unipolar LBBAP, 10 patients demonstrated a right bundle branch block (RBBB) pattern with a paced QRSd of 123.0 ± 5.7 ms (*P* = 0.001 vs. baseline) (Fig. 2b). The LBB potential could be recorded in 7 patients from the LBB lead (70.0%). The LVAT for all LBBAP patients was 72.5 ± 9.4 ms, and the R wave amplitude, pacing impedance, and unipolar pacing capture threshold were 9.9 ± 7.2 V, 678 ± 102 Ω, and 0.84 ± 0.17 V/0.4 ms, respectively.

During the procedure, intraoperative BVP resulted in a significant reduction in the QRSd from 158.0 ± 13.0 ms at baseline to 132.0 ± 4.5 ms (*P* = 0.019). Compared with BVP, unipolar LBBAP resulted in a right bundle branch block pattern (Fig. 2b) and further reduction of the QRSd to 123.0 ± 5.7 ms (*P* = 0.006 vs. baseline and *P* = 0.021 vs. BVP). After device implantation, and when LOT-aCRT implanted and worked, a LBBB correction pattern was visible (Fig. 2c) and the QRSd was further shortened to 121.0 ± 3.8 ms, but compared with LBBAP, there was no statistical significance (*P* > 0.05).

In group 2, intraoperative BVP resulted in a significant reduction in the QRSd from 176.7 ± 19.7 ms at baseline to 133.3 ± 8.2 ms (*P* = 0.011). However, compared with the 121.0 ± 3.8 ms from LOT-aCRT in group 1, BVP in group 2 did not have any advantage in reducing the QRSd and the difference was statistically significant (*P* < 0.01, Table 3).

**Table 3:** Characteristics during a follow-up period of 9 months in patients implanted with a CRT-D/P (mean ± SD) (n = 21)

		Total (n = 21)	Group 1 (n = 10)	Group 2 (n = 11)	*P* value
LAD (mm)	Before procedure	3.36 ± 0.50	3.4 ± 0.55	3.3 ± 0.52	0.840
	12 months after procedure	2.45 ± 0.52	2.4 ± 0.55	2.5 ± 0.55	0.770
	*P* value	0.000	0.032	0.024	–
LVEDD (mm)	Before procedure	65.1 ± 9.1	43.6 ± 5.4	42.6 ± 5.3	0.336
	12 months after procedure	58.7 ± 10.2	39.8 ± 4.1	45.2 ± 8.7	0.303
	*P* value	0.319	0.030	0.060	–
LVEF (%)	Before procedure	55.4 ± 8.7	55.0 ± 5.1	55.8 ± 12.0	0.894
	12 months after procedure	53.1 ± 3.0	52.0 ± 4.2	54.0 ± 1.3	0.302
	*P* value	0.002	0.011	0.143	–

*LVEDD* left ventricular end diastolic diameter, *LVEF* left ventricular ejection fraction, *NT-proBNP* N-terminal pro B type brain natriuretic peptide; *NYHA* New York Heart Association; *QRSd* QRS duration, *VT* ventricular tachycardia, *VF* ventricular fibrillation

As the aCRT algorithm provides mostly LV only pacing (which means LBBAP in group1 and CS pacing in group 2) in patients with preserved AV conduction, the percentage of LV only pacing in the aCRT arm was high: 70.5% in group 1 and 72.8% in group 2.

### Follow-up

The mean follow-up time was 574 ± 188 days. At baseline, the two groups were matched for follow-up time (572 ± 207, 575 ± 190 days, *P* > 0.05). Among all 21 patients, the CS lead parameters were stable during follow-up. In group 1, the LBBAP capture threshold, R-wave amplitude, and lead impedance were 0.74 ± 0.25 V, 13.36 ± 5.23 mV, and 533.73 ± 32.31 Ω during the 9-month follow-up (all *P* > 0.05, respectively, between the time of device implantation and the follow-up visit). In group 2, the RV lead parameters were also stable during follow-up. No patients showed signs of dislodgement, loss of capture, infection, embolism, or stroke associated with the implantation. The ventricular pacing rate was 95%. There were 8 VT/VF episodes treated with antitachycardia pacing that had an electrogram available for adjudication (3 episodes in group 1, 5 episodes in group 2). However, the rate of VT/VF therapy was not significantly different (*P* = 0.175) between the two groups.

Transthoracic echocardiogram (Fig. 3b) evaluation data at baseline and at the 3-month and 9-month follow-ups were available in all 21 patients receiving successful aCRT. As shown in Table 3, the symptoms and the median NYHA classification score improved significantly, with the latter decreasing from 3.36 ± 0.50 to 2.45 ± 0.52 (*P* = 0.016). LVEF (33.9 ± 3.9% vs. 45.4 ± 8.7%, *P* = 0.002) and NT-proBNP (2937 ± 1646 vs. 1832 ± 1541, *P* = 0.014) significantly improved at the follow-up visit. LVEDD (65.1 ± 9.1 mm vs. 58.7 ± 10.2 mm, *P* = 0.319) was improved at the 9-month follow-up visit, but the improvement was not significant.

Compared to the baseline, patients in group 1 showed significant improvement in LVEF and NT-proBNP levels, while patients in group 2 showed a trend of improvement in these parameters (Table 3).

## Discussion

### Major findings

We studied a new technique using adaptive left bundle branch–optimized cardiac resynchronization therapy (LBBAP combined with coronary venous pacing) in a cohort of patients with LBBB and HF. The principal findings were as follows. (1) LOT-aCRT was feasible in a small nonrandomized patient cohorts with reduced LVEF and LBBB. (2) After device implantation, LOT-aCRT was superior to BV-CRT, associated with shorter paced QRSd and shorter LVAT. (3) LOT-aCRT resulted in significant improvements in clinical NYHA and LVEF assessments during the follow-up period of 9 months. (4) There were no major implantation-related adverse events during the peri-operative period or follow-up.

### Anatomical definition

CRT using BVP is an integral part of therapy for patients with HF that involves reduced LVEF and BBB, particularly LBBB [28]. However, up to one-third of patients treated with BVP-CRT are still considered nonresponders [3]. The reasons for BVP-CRT nonresponse are many but include LV scar burden and distribution, a suboptimal LV stimulation site, sex, and limited electrical or mechanical dyssynchrony [4]. Patients with ICM experience a similar BVP-CRT response rate to their nonischaemic counterparts [29]. However, a higher overall scar burden, a larger number of severely scarred segments, and greater scar density near the LV lead tip portend an unfavourable response to BVP-CRT in ICM patients [30]. There is evidence that CRT is not salutary in patients with posterolateral scarring [5].

A substudy of the aCRT trial revealed that patients with a high percentage of adaptive LV pacing showed better clinical improvement and PQ intervals than patients within the normal range [31]. The mechanism of benefit in this patient cohort was speculated to be “fusion” of the excitation from LV pacing with intrinsic conduction propagating through the still preserved His-Purkinje system [32].

### Electrophysiological definition

Permanent LBBAP is an effective form of physiologic pacing with high success rates in patients with intact His-Purkinje conduction [4]. LBBAP can serve as a new CRT technique to correct LBBB, provide ventricular synchrony, and improve clinical symptoms with reverse remodelling of the LV [7].

There is the evidence that LV activation time is only minimally increased in RBBB but significantly increased in LBBB [33]. During unipolar LBBAP, as RV is predominantly activated via myocardial conduction, more RV dyssynchrony may be present than in HBP. However, it does not causes LV dyssynchrony since LV activation occurs via the His-Purkinje system. Therefore, in patients undergoing permanent LBBAP, synchronization of delayed RV activation and normal LV activation is feasible.

### The Advantages of LOT-aCRT

The aCRT algorithm is a novel algorithm that periodically measures intrinsic conduction and dynamically adjusts CRT pacing parameters as needed [12]. Therefore ambulatory adjustment of the pacing configuration could provide LV pacing only or BVP. Based on periodic automatic evaluation of electrical conduction, the aCRT algorithm provides RV-synchronized LV pacing when AV conduction is normal or BV pacing when AV conduction is prolonged [15].

In group 1, the CS lead was connected to the pace-sensing portion of the RV port, and the LBBAP lead was connected to the LV port. Therefore, LV pacing means LBBAP pacing, while RV pacing means CS pacing. When the PR interval is normal, aCRT provides LBBAP only, while for a long PR interval, it provides BV pacing.

In patients with LBBB, only LBBAP might have been sufficient to resynchronize the LV. However, LBBAP achieved only partial reduction of the QRSd in those patients with a baseline surface ECG of atypical LBBB morphology [11]. Intra- or interventricular dyssynchrony cannot be reduced through LBBAP. LOT-aCRT offers the advantage of using the LV lead in addition to LBBAP in a potential scenario in which conduction disease progresses. A previous study demonstrated the efficacy of aCRT in patients with preserved AV conduction [31].

Group 2 had only an 18.8% reduction in QRSd, but a previous study described a 25% reduction with CRT in LBBB and synchronous AV conduction [34]. These patients were insufficiently optimized and LOT-aCRT may have been better suited to their treatment.

In patients with LBBB and cardiomyopathy, LOT-aCRT resulted in significant electrical resynchronization. In group 1 of our study, 62.5% of whose subjects had severe ischaemic cardiomyopathy, LOT-aCRT resulted in a significantly greater reduction of the QRSd to 121.0 ± 3.8 ms from 158.0 ± 13.0 ms and high clinical and echocardiographic response rates. Our results indicated that patients with LBBB and a higher overall scar burden might be the ideal candidates for LOT-aCRT.

The device connection of this study may not be commonly utilized. Moreover, the CS lead was connected to the pacing-sense portion of the RV. It is not clear whether the sensing of the CS lead may potentially lead to malfunction of defibrillation.

### Limitations

First, LOT-aCRT is time consuming. The duration of the operation was 152 ± 31 min, and the duration of X-ray fluoroscopy was 29.2 ± 8.8 min; both were longer than those stated in a previous report (117 ± 48 and 16.4 ± 12.3 min) [4] and those in the control group. Second, this study included only a small sample from a single centre. Third, this study had a short follow-up interval, although we expect favourable long-term clinical benefits. Fourth, the device connection of this study may not be commonly utilized. Furthermore, this study enrolled only 11 ischaemic patients. Although this study does not provide sufficient data to support a general conclusion, we observed significant echocardiographic and clinical improvement in these HF patients treated with LOT-aCRT.

## Conclusions

The study demonstrates that LOT-aCRT is clinically feasible in patients with systolic HF, LBBB and preserved AV conduction. LOT-aCRT was associated with significant reduction in QRS duration and improvement in LV function.

## Data Availability

Data are available from the corresponding author upon reasonable request due to privacy or other restrictions.

## Abbreviations

CRT: Cardiac resynchronization therapy
BVP: Biventricular pacing
LBBAP: Left bundle branch area pacing
QRSd: QRS duration
HBP: His bundle pacing
LOT-CRT: LBB-optimized LV pacing
HF: Heart failure
LVEF: Left ventricular ejection fraction
LV: Left ventricle
RAO: Right anterior oblique
LAO: Left anterior oblique
UTP: Unipolar tip pacing
LVAT: Left ventricular activation time
ICD: Implantable cardioverter-defibrillator
LBBB: Left bundle branch block
RBBB: Right bundle branch block
RV: Right ventricle
ICM: Ischaemic cardiomyopathy
CRT-D: CRT-defibrillator
CRT-P: CRT-pacemaker
ms: msec
