# Correction: Effects of adaptive left bundle branch–optimized cardiac resynchronization therapy: a single centre experience

**DOI:** 10.1186/s12872-022-02978-y

**Published:** 2022-12-22

**Authors:** Xiang-Fei Feng, Ling-Chao Yang, Yan Zhao, Yi-Chi Yu, Bo Liu, Yi-Gang Li

**Affiliations:** Department of Cardiology, School of Medicine, Xinhua Hospital, Shanghai Jiao Tong University, #1665, KongJiang Road, Shanghai, 200092 China

**Correction: BMC Cardiovascular Disorders (2022) 22:360** **https://doi.org/10.1186/s12872-022-02742-2**

Following publication of the original article [1], the author has changed the table 3 and it should read as follows:

**Table:** Characteristics during a follow-up period of 9 months in patients implanted with a CRT-D/P (mean ± SD) (n = 21)

		Total (n = 21)	Group 1 (n = 10)	Group 2 (n = 11)	*P* value
NYHA classification score	Before procedure	3.36 ± 0.50	3.4 ± 0.55	3.3 ± 0.52	0.840
	3 Months after procedure	2.54 ± 0.52	2.6 ± 0.55	2.5 ± 0.55	0.770
	9 Months after procedure	2.45 ± 0.52	2.4 ± 0.55	2.5 ± 0.55	0.770
	*P* value	0.000	0.032	0.024	–
LVEDD (mm)	Before procedure	65.1 ± 9.1	68.2 ± 12.3	62.6 ± 5.3	0.336
	3 Months after procedure	63.4 ± 10.1	64.4 ± 12.6	62.4 ± 8.3	0.781
	9 Months after procedure	58.7 ± 10.2	62.2 ± 11.3	55.2 ± 8.7	0.303
	*P* value	0.319	0.735	0.229	–
LVEF (%)	Before procedure	33.1 ± 3.0	32.0 ± 4.2	34.0 ± 1.3	0.302
	3 Months after procedure	40.9 ± 7.0	41.6 ± 7.5	40.3 ± 7.3	0.782
	9 Months after procedure	45.4 ± 8.7	45.0 ± 5.1	45.8 ± 12.0	0.894
	*P* value	0.002	0.011	0.143	–
QRSd (ms)	Before procedure	168.2 ± 18.9	158.0 ± 13.0	176.7 ± 19.7	0.104
	9 Months after procedure	131.4 ± 15.5	121.0 ± 3.8	133.3 ± 8.2	0.001
	*P* value	0.001	0.005	0.011	–
NT-ProBNP (pg/ml)	Before procedure	2937 ± 1646	3240 ± 2258	2684 ± 1083	0.634
	3 Months after procedure	1832 ± 1541	1151 ± 1774	2066 ± 1444	0.607
	*P* value	0.014	0.04	0.219	–
VT/VF episodes (n)		8	3	5	0.175
Follow-up period (d)		574 ± 188	572 ± 207	575 ± 190	0.981

*LVEDD* left ventricular end diastolic diameter, *LVEF* left ventricular ejection fraction, *NT-proBNP* N-terminal pro B type brain natriuretic peptide, *NYHA* New York Heart Association, *QRSd* QRS duration, *VT* ventricular tachycardia, *VF* ventricular fibrillation

Also, the baseline QRSd value were changed from 33.9 ± 3.9% to 33.1 ± 3.0%.

So, the text under "Baseline characteristics" will read as follows:

 The echocardiographic indices, including LVEF, LVEDD, NYHA classification, and NT-proBNP are shown in Table 3. Baseline parameters were similar between the two groups (all *P* > 0.05). The baseline LVEF and the baseline QRSd (Fig. 2a) were 33.1 ± 3.0% and 168.2 ± 18.9 ms, respectively. At baseline, the QRSd of the two groups were matched (158.0 ± 13.0, vs. 176.7 ± 19.7, *P* > 0.05).

Also, text under "Follow‑up" will read as follow: Transthoracic echocardiogram (Fig. 3b) evaluation data at baseline and at the 3-month and 9-month follow- ups were available in all 21 patients receiving successful aCRT. As shown in Table 3, the symptoms and the median NYHA classification score improved significantly, with the latter decreasing from 3.36 ± 0.50 to 2.45 ± 0.52 (*P* = 0.016). LVEF (33.1 ± 3.0% vs. 45.4 ± 8.7%, *P* = 0.002) and NT-proBNP (2937 ± 1646 vs. 1832 ± 1541, *P* = 0.014) significantly improved at the follow-up visit. LVEDD (65.1 ± 9.1 mm vs. 58.7 ± 10.2 mm, *P* = 0.319) was improved at the 9-month follow-up visit, but the improvement was not significant.

The original article has been corrected.

